# Development of Indirect Competitive ELISA and Visualized Multicolor ELISA Based on Gold Nanorods Growth for the Determination of Zearalenone

**DOI:** 10.3390/foods10112654

**Published:** 2021-11-02

**Authors:** Tianyu Ma, Kaixin Liu, Xiao Yang, Jingying Yang, Mingfei Pan, Shuo Wang

**Affiliations:** 1 State Key Laboratory of Food Nutrition and Safety, Tianjin University of Science & Technology, Tianjin 300457, China; maty1128@126.com (T.M.); Liukx2019@163.com (K.L.); yangx2021@126.com (X.Y.); yangjy0823@126.com (J.Y.); s.wang@tust.edu.cn (S.W.); 2 Key Laboratory of Food Nutrition and Safety, Ministry of Education of China, Tianjin University of Science and Technology, Tianjin 300457, China

**Keywords:** zearalenone, gold nanorods, indirect competitive ELISA, visualized multicolor ELISA

## Abstract

In this study, a zearalenone (ZEN) hapten was designed and prepared against the mycotoxin ZEN, and the original coating ZEN-ovalbumin (ZEN-OVA) was prepared by conjugation with OVA. Based on the gold nanorods (AuNRs) of uniform size and stable properties synthesized by the seed-mediated method, the indirect competitive enzyme-linked immunosorbent assay (ic-ELISA) and the AuNRs growth-based multicolor ELISA for detecting ZEN toxin were further established. Under the optimal experimental conditions, the coating amount of ZEN-OVA: 0.025 μg/well, antibody (Ab) dilution factor: 32,000 times, blocking solution: 0.5% skimmed milk powder, enzyme-labeled secondary Ab diluted 10,000 times, and a pH of the PBS buffer at 7.4, the sensitivity (*IC*_50_) of the established ic-ELISA for ZEN detection reached 0.85 ± 0.04 μg/L, and the limit of detection (*IC*_15_) reached 0.22 ± 0.08 μg/L. In the multicolor ELISA based on the growth of AuNRs, as the content of ZEN increased, the mixed solution exhibited a significant color change from brownish red to colorless. ZEN concentration as low as 0.1 μg/L can be detected by the naked eye (brown red to dark gray). This study provided an effective analysis strategy for the rapid screening and accurate monitoring of the ZEN contaminant in foods.

## 1. Introduction

Zearalenone (ZEN) is a nonsterol estrogen mycotoxin mainly produced by *Fusarium* genera, which is widely found in grain crops, such as corn, wheat, and barley [1,2]. The degree of contamination of food crops by *Fusarium* mainly depends on the moisture and temperature conditions; the optimal growth temperature for *Fusarium* is between 24 and 32 °C and the optimal humidity is 40% [3]. Therefore, in areas with sufficient rainfall and high relative humidity, cereals are likely to be contaminated with *Fusarium* in all steps of production, storage, and processing [4]. ZEN can cause excessive estrogen syndrome in animals such as pig, poultry, and humans, as well as immunotoxicity, genotoxicity, and suspected carcinogenicity [5,6]. It has been reported that the potential etiological mechanism of breast cancer involves changes in the cytochrome P450 (CYP) enzyme, which is related to ZEN [7]. Yu et al. have demonstrated that the ZEN on *2,3,7,8*-Tetrachlorodibenzo-*p*-dioxin (TCDD)-induced CYP1A1 activity and gene expression involved the estrogen receptor pathway [8]. In addition, due to its similar structure to endogenous estrogen, ZEN can show estrogen activity in vivo and competitively bind with estrogen receptors, thus affecting estrogen secretion in humans or animals, resulting in reproductive organ abnormalities, infertility, abortion, and other diseases [9,10,11]. At present, many countries and organizations have regulated the maximum residue levels (MRLs) of ZEN in foods, although a consistent standard has not yet been obtained (European Union: 20–400 μg/kg in different types of foods, Russia: 1000 μg/kg in hard wheat, flour and wheat germ; China: 60 μg/kg in cereals and its products) [12,13,14]. Therefore, the development of effective strategies for ZEN detection in foods is of great significance for protecting the health of humans and animals.

Currently, traditional instrumental methods based on liquid chromatography (LC), gas chromatography (GC), and mass spectrometry (MS) have been widely applied in the detection of ZEN contaminant in foods and animal feed samples [15,16,17,18,19,20]. However, these techniques require sophisticated instruments, professional and experienced operators, and long test time, which limits the practical application to a certain extent. Engvall and Perlmann achieved the quantitative detection of a solid phase enzyme immunoassay for the first time, marking the successful construction of an enzyme linked immunosorbent assay (ELISA) [21,22]. The ELISA has the characteristics of simple operation, high specificity, and low cost, and has gradually become the mainstream technology in the field of rapid detection [23,24]. Improving the sensitivity and lowering the detection limit of ELISA can promote the further application of this effective technology [25,26]. Signal amplification has been demonstrated by many researchers to improve the sensitivity of ELISA. In particular, the introduction of various nanomaterials has further improved the performance of traditional ELISA. For example, Zhang et al. synthesized CdTe/CdS/ZnS quantum dots (QDs) in the aqueous phase and developed a fluorescent immunoassay (FLISA) for detecting ZEN in corn [27]. Xiong et al. reported an advanced enzyme-assisted etching method in which gold nanorods (AuNRs) were applied as the signal carrier for aflatoxin B_1_ (AFB_1_) in corn samples to amplify the ELISA signal [28]. Liu et al. constructed a horseradish peroxidase (HRP)-mediated ratio fluorescence ELISA based on gold and silver bimetallic nanoclusters (Au-AgNCs) to detect zearalenone, which significantly improved the detection limit [29].

**Scheme 1:**
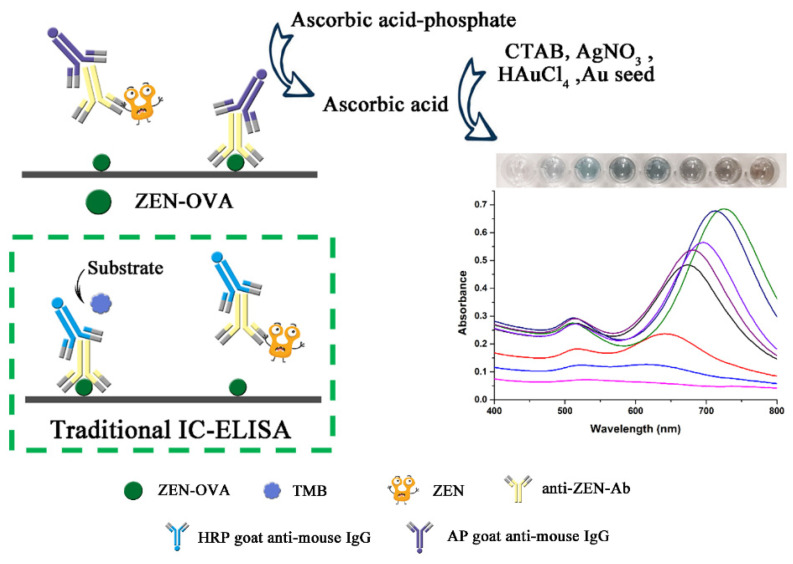
Procedure of the visualized AuNRs growth-based multicolor ELISA for ZEN detection compared with the traditional ic-ELISA.

In this study, we successfully prepared the ZEN hapten and ZEN coating antigen (ZEN-ovalbumin (ZEN-OVA)) and developed an indirect competitive-ELISA (ic-ELISA) strategy using monoclonal antibodies (anti-ZEN-Abs) with high sensitivity and specificity. On this basis, a visual multicolor ELISA was developed based on alkaline phosphatase (AP) converting ascorbic acid–phosphate (VcP) to ascorbic acid (Vc) to control the growth of AuNRs. Compared with traditional ic-ELISA for ZEN, the visualized multicolor ELISA based on AuNRs growth offered a more convenient and intuitive strategy for the detection of ZEN contaminant (Scheme 1).

## 2. Materials and Methods

### 2.1. Material and Apparatus

Hexadecyl trimethyl ammonium bromide (CTAB, 99%) was purchased from Solarbio (Beijing, China). Chloroauric acid (HAuCl_4_) for the synthesis of AuNRs was obtained from Sigma-Aldrich (St. Louis, MO, USA). Silver nitrate (AgNO_3_), Vc, VcP, and sodium borohydride (NaBH_4_) for the synthesis of AuNRs, *1*-ethyl-*3*-[*3*-(dimethylamino) propyl] carbodimide (EDC) and OVA for the synthesis of the conjugate of ZEN-OVA were obtained from the Sinopharm Chemical Reagent Co., Ltd. (Shanghai, China). Pyridine and carboxymethoxylamine (CMO) were purchased from TCI Development Co. Ltd. (Shanghai, China). The anti-ZEN monoclonal antibody (anti-ZEN-Ab, 1.0 mg/mL) was purchased from Shandong Lvdu Biotechnology Co. Ltd. (Shandong, China). *3*,*3*,*5*,*5*-tetramethylbenzi-dine (TMB), HRP goat anti-mouse IgG conjugated (1.0 mg/mL), AP goat anti-mouse IgG conjugated (3.5 mg/mL), AFB_1_, ZEN and the structural analogues (α-zearalenol, α-zearalanol, β-zearalenol, zearalanone, β-zearalanol) (1.0 mg/L) were also purchased from Sigma-Aldrich (St. Louis, MO, USA). T-2 toxin, ochratoxin A (OTA), and fumonisin B_2_ (FB_2_) were purchased from Toronto Research Chemicals (Toronto, ON, Canada).

The microplate reader for reading the absorbance values was purchased from Thermo Fisher Scientific (Waltham, MA, USA). The UV-visible spectrophotometer (Cary 50 Bio) was obtained from Varian (Salt Lake, CA, USA). The 96-well polystyrene microplates, multichannel pipettes (100–300 μL), and single-channel pipettes (2.5–1000 μL) were obtained from Thermo Fisher Scientific (Waltham, MA, USA). The transmission electron microscopy (TEM) images were obtained from Talos G2 200X electronic microscope (Thermo Fisher Scientific (Waltham, MA, USA). A vortex machine (HQ-60) was purchased from North Tongzheng Biotechnology Development Company (Beijing, China). Milli-Q Ultrapure Water System was purchased from Milli-Q Millipore, (Bedford, MA, USA).

### 2.2. Preparation of the Coating Antigen—The Conjugate of ZEN-OVA

The preparation method of ZEN-CMO was modified according to the previous literature [30]. Briefly, 10.0 mg of ZEN was dissolved in 200 μL of methanol and mixed well. Then, 20.0 mg of CMO and 1.0 mL of anhydrous pyridine were sequentially added into the above mixed solution. The mixture was stirred and reacted under nitrogen protection for 24 h. After the reaction was completed, the mixture was placed in a vacuum oven at 80 °C to remove pyridine and store at 4 °C for later use. We mixed 9.78 mg of the above conjugate ZEN-CMO and 14.0 mg of EDC, successively dissolved it in 1.0 mL of DMF, and stirred and activated it overnight at 4 °C. In total, 10.0 mg of OVA was dissolved in 2.0 mL of NaHCO_3_ (130 mmol/L) and precooled. The activated product was added dropwise under ice bath conditions. Two hours later, the product was placed under 4 °C overnight and dialyzed against PBS buffer solution for 72 h and stored at −20 °C.

### 2.3. Test Procedure of ic-ELISA

The antigen ZEN-OVA was dissolved in the coating solution and mixed evenly. The mixture was added to a microplate (0.025, 0.05, 0.1 μg/well, 100 μL/well), and incubated overnight at 4 °C. After washing the plate with PBST (0.01 mol/L PBS and 0.1% Tween-20) 3 times, the blocking solution (PBS containing 0.5% skimmed milk powder, 200 μL/well) was added, and we incubated the mixture at 37 °C for 1 h. After washing, the mixture of ZEN standards were diluted to different concentrations (50 μL) and anti-ZEN Ab (50 μL) was added and reacted at 37 °C for 1 h. Then, 100 μL of HRP goat anti-mouse IgG was added into each well and incubated for 0.5 h. After washing the plate with PBST 5 times, 100 μL of the TMB substrate solution was added into each well. After incubation for 15–30 min, the reaction was terminated with 50 μL of H_2_SO_4_ (1.25 mol/L). Then, the absorbance was measured at 450 nm using a microplate reader.

### 2.4. Specificity of ic-ELISA

The specificity of Abs is related to the structure of the antigenic determinant and is expressed as the cross-reactivity rate. Ten mycotoxins including ZEN, five structural analogs of ZEN (α-zearalenol, β-zearalenol, α-zearalanol, β-zearalanol, and zearalanone), and four common mycotoxins (AFB_1_, OTA, T-2 toxin, and FB_2_) were used as competing standards to determine the cross-reactivity rate to anti-ZEN-Ab by ic-ELISA. The mentioned analytes were diluted with PBS to the following concentrations (1000, 333.3, 111.11, 37.03, 12.34, 4.11, 1.37, 0.45, 0.15, 0.05, and 0.016 μg/L). The following equation was used to calculate the cross-reaction rate.

CR (%) = IC_50_ (50% inhibitory concentration (ZEN))/IC_50_ (50% inhibitory concentration (competitor)) × 100% (1)

### 2.5. AuNR Growth-Based Multicolor ELISA for ZEN

**Preparation ofAuNRs seed liquid.** The AuNRs were synthesized by the seed-mediated method in this study. A total of 1.0 mL of CTAB (0.2 mol/L) and 1.0 mL of HAuCl_4_ (0.5 mmol/L) were added into a round-bottom flask and mixed thoroughly. Then, 120 μL of NaBH_4_ (0.01 mol/L) was added and stirred gently for 2 min. The obtained mixture was used as the seed liquid for AuNR growth.

**Effect of Vc dosage on AuNR growth.** The mixed solution containing 125 μL of CTAB (0.2 mol/L), 1.5 μL of AgNO_3_ (0.01 mol/L), and 12.5 μL of HAuCl_4_ (0.01 mol/L) was added to each well of the enzyme-labeled plate. After mixing, the Vc (0.01 mol/L) solution of different volumes (0–20 μL) was added to control the total volume of the solution to 240 μL. Next, 10 μL of the seed solution was added to mediate the production of AuNRs. After mixing evenly, the mixture was incubated at room temperature for 1 h, and UV absorption spectra of the grown AuNRs were recorded.

**Procedure of visualized multicolor ELISA based on AuNR growth.** The encapsulation process of the ZEN-OVA conjugate and its competitive binding with anti-ZEN Abs were the same as ic-ELISA. The difference was that 100 μL of AP-goat anti-mouse IgG diluted 10,000 times with Tris-HCl buffer (1 mmol/L pH 7.4) was added to each well. After incubation at 37 °C for 30 min and full washing, 80 μL of VcP (15 mmol/L) was added into each well. After full mixing, the mixture was incubated at 37 °C for 1 h. Then, 50 μL of the above reaction solution was mixed evenly with the solution containing 125 μL of CTAB (0.2 mol/L), 1.5 μL of AgNO_3_ (0.01 mol/L), and 12.5 μL of HAuCl_4_ (0.01 mol/L) and controlled to 240 μL with ultrapure water. After 10 µL of seed solution was added, the mixture was incubated at room temperature for 1 h and tested by naked eye and UV absorption spectroscopy.

## 3. Results and Discussion

### 3.1. Preparation and Characterization of ZEN-CMO and ZEN-OVA Conjugate

In this study, the prepared ZEN-CMO (Mr: 391.28) was analyzed by mass spectrometry (Figure S1). In an anionic environment, the characteristic ion peaks of ZEN-CMO [M-1]¯ at 390.40 and [2M-1]^−^ at 781.80 were observed, proving the successful preparation of the hapten ZEN-CMO with acceptable purity. The ZEN-CMO product was further conjugated with OVA to obtain ZEN-OVA conjugate, the concentration of which was determined to be 3.85 mg/mL by a commercial BCA kit. Furthermore, the prepared ZEN-OVA conjugate was tested for its conjugate ability to anti-ZEN Abs (Table S1). When the Abs was diluted 16,000 times, the *OD*_450_ value reached 1.253, and the inhibition rate reached 98.92%, indicating the successful coupling of ZEN-CMO and OVA, and the obtained ZEN-OVA conjugate could be used for subsequent immunoassay experiments.

### 3.2. Conditions Optimization of Traditional ELISA

**Dilution times of ZEN-OVA conjugate and anti-ZEN Abs.** Different amounts of ZEN-OVA conjugate were coated on a 96-well microtiter plate to optimize the dilution times through ic-ELISA. According to the results shown in Table S2, when the coating amount of the ZEN-OVA conjugate was 0.1 μg/well and the Ab dilution factor was 64,000, the OD_450_ value reached 0.858, and the IC_50_ was 2.68 μg/L. When the coating amount was 0.05 μg/well and 0.025 μg/well, the dilution factor was 32,000, the corresponding OD_450_ and IC_50_ values were 1.078 and 0.793, 1.06 μg/L and 0.85 μg/L, respectively. It can be clearly seen that the IC_50_ increased with the increase in the coating amount of ZEN-OVA, which was because the excessive amount of ZEN-OVA bound to the Ab led to the decrease in detection sensitivity. When the coating amount was 0.025 μg/well and the Ab dilution factor was 32,000, the color development was relatively stable and the IC_50_ reached the lowest value, which was selected as the optimal condition for subsequent experiments.

**Blocking Solution.** The blocking solution was applied to block the excess binding sites in the micropores. A higher concentration of the blocking solution may affect the subsequent binding between the antigen and Ab, thereby reducing the sensitivity of the method. In the study, the IC_50_ values of the ic-ELISA method using different concentrations (0.5% and 1%) of skimmed milk powder as the blocking solution were compared (Table S3). The 0.5% skimmed milk powder had an IC_50_ of 0.85 μg/L, less than using 1% skimmed milk powder (2.41 μg/L), which was selected as the blocking solution.

**pH value of PBS diluent.** PBS buffers with different pH values (5.7, 7.4, and 8.5) were used for Ab dilution and ic-ELISA tests were performed (Table S4). When PBS was used at pH 7.4, the IC_50_ value was only 0.85 μg/L, which was significantly lower than that obtained at pH 5.7 and 8.5 (2.95 μg/L and 1.86 μg/L). At this time, the corresponding absorbance value (λ = 450 nm) was also significantly higher than the other two pH values, because the acidic or alkaline environment affected the binding reaction between the antigen and Ab as well as enzyme-labeled antibody, resulting in the reduction in detection sensitivity. Therefore, a PBS buffer of pH 7.4 was chosen as a diluent for Ab and ZEN standards.

### 3.3. ic-ELISA Standard Curve

**Figure 1:**
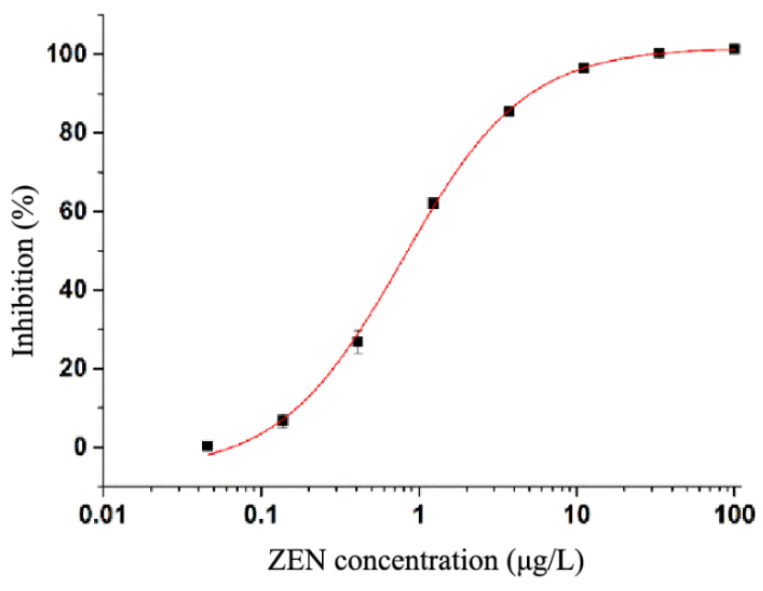
Standard curve of ZEN by ic-ELISA.

Under the optimal conditions: coating amount of ZEN-OVA 0.025 μg/well, Ab dilution 32,000 times, blocking solution 0.5% skim milk powder in PBS, PBS buffer (pH 7.4) as diluent, the standard curve of ic-ELISA for ZEN is shown in Figure 1. The sensitivity (IC_50_) and limit of detection (IC_15_) reached 0.85 ± 0.04 μg/L and 0.22 ± 0.08 μg/L, indicating that this method can provide accurate and sensitive analysis for the ZEN toxin.

### 3.4. Specificity of Traditional ELISA

In order to evaluate the specificity of the established ic-ELISA method to ZEN, five ZEN structural analogs (α-zearalanol, β-zearalanol, α-zearalenol, β-zearalenol, and zearalenone) and four common mycotoxins (AFB_1_, OTA, FB_2_, and T-2 toxin) were selected and analyzed (Table 1). The cross-reaction rate of four structural analogs α-zearalanol, β-zearalanol, α-zearalenol, and β-zearalenol were 35.27%, 45.70%, 29.72% and 17.93%, respectively; the cross-reaction rate of zearalenone was only 1.58%; and there was no obvious cross-reaction with other mycotoxins. These results proved that the established ic-ELISA method had high specificity.

### 3.5. Multicolor ELISA Based on AuNRs Growth

**Figure 2:**
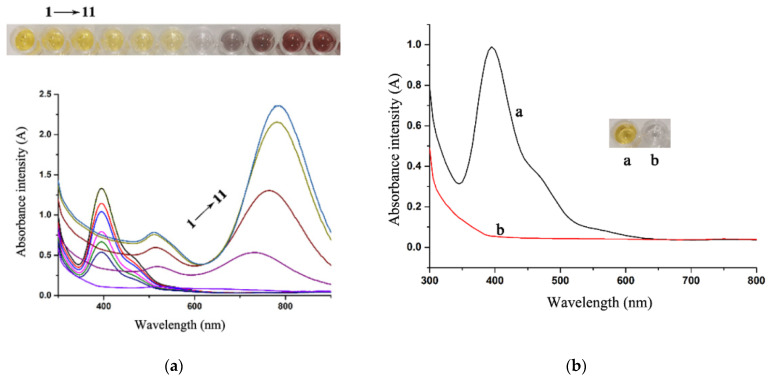
(**a**) UV-visible absorption spectra of AuNRs grown with different volumes of Vc; (**b**) UV-vis spectra and visual results of the AuNRs growth solution before and after adding excessive VcP (a: before; b: after).

The AuNRs used in the study were prepared by a traditional seed-mediated method. The amount of Vc had a very important effect on the morphology and properties of the prepared AuNPs. Figure 2a shows the results of AuNRs obtained under different Vc solution additions. When a small amount of Vc solution was added (No. 1–6), the mixed solution appeared yellow, and the corresponding UV-visible absorption spectrum had a significant absorption peak at 400 nm, which was the characteristic absorption of HAuCl_4_. With the increase in Vc solution volume, the color of the solution gradually lightened. When the added amount was 12.0 μL, the solution was colorless by naked eye observation. Meanwhile, no significant absorption was observed in the wavelength range greater than 500 nm. This was because when the amount of Vc was low, only Au(III) was reduced to colorless Au(I) (No. 7). When the amount of Vc further increased, a new and gradually enhanced UV absorption peak was formed at 500–800 nm, indicating that AuNRs gradually grew (No. 8–11), which also proved that the Vc supplemental level played a very important role in regulating AuNRs growth.

**Figure 3:**
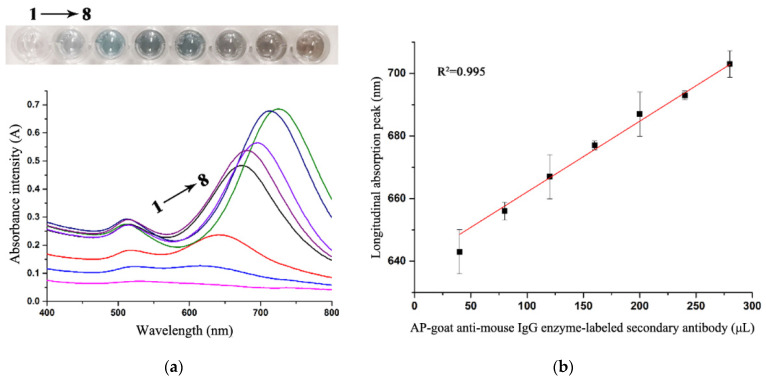
(**a**) UV-visible absorption spectra of AuNRs grown with different volumes of AP-labeled secondary Ab; (**b**) the standard curve of AP-labeled secondary Ab and longitudinal absorption peaks of AuNRs.

AP can induce the conversion of VcP to Vc and then promote the growth of AuNRs. Figure 2b shows the UV absorption spectra and visual results of the AuNR growth solution before and after the addition of excessive VcP. It was clearly observed that the addition of VcP changed the growth solution from yellow (curve a) to colorless (curve b). In addition, when excessive VcP was added, there was no absorption peak in the wavelength range of 500–800 nm, indicating that VcP could also reduce Au (III) to Au (I) but could not promote the growth of AuNRs. It was confirmed that the conversion of VcP to Vc by AP was a necessary prerequisite for promoting AuNR growth. Furthermore, different amounts of AP-labeled secondary Abs were mixed with a fixed amount of Vc sodium phosphate and incubated at a certain temperature to generate different amounts of Vc, which then produced AuNRs with different aspect ratios, colors, and UV absorption spectrum, which can be used as the basis for visual detection. Figure 3 shows the effect of adding different volumes of AP-goat anti-mouse IgG enzyme-labeled secondary Ab on the growth of AuNRs. With the increase in the amount of enzyme-labeled secondary Ab, the color of the mixed solution gradually became darker, from colorless to reddish brown. The UV absorption spectra showed that the longitudinal absorption peak of the prepared AuNRs had a significant red shift. There was a good linear relationship between the amount of AP goat anti-mouse IgG conjugated secondary Ab (40–80 μL) and the longitudinal absorption peak of the AuNRs produced (R^2^ = 0.995) (Figure 3b).

**Figure 4:**
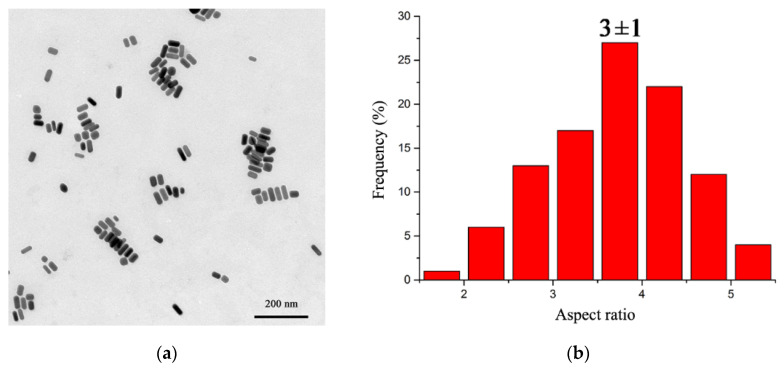

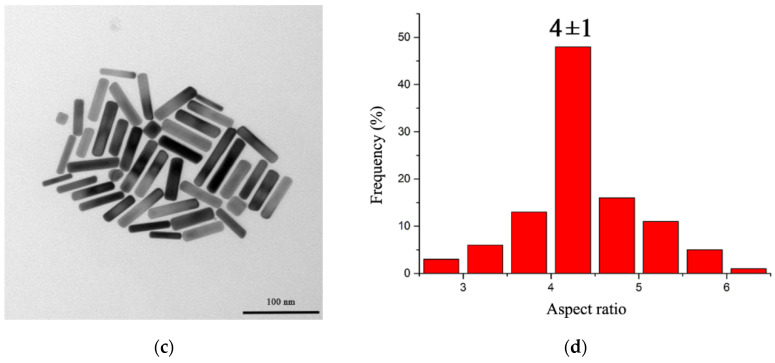
TEM images and aspect ratio of particle size analysis of AuNRs prepared by adding 120 (**a**,**b**) and 280 μL (**c**,**d**) of enzyme-labeled secondary Abs.

Transmission electron micrographs (TEM) of AuNRs prepared with the addition of 120 and 280 μL of enzyme-labeled secondary Abs are shown in Figure 4. The size of the AuNRs prepared with 120 μL of enzyme-labeled secondary Ab were uniform and moderate, with an average aspect ratio of 3.5 (Figure 4a,b). When 280 μL of enzyme-labeled secondary Ab was used, the aspect ratio of AuNRs increased to 4.5 (Figure 4c,d). The results showed that the addition of AP goat anti-mouse IgG enzyme-labeled secondary Ab had a direct effect on the increase in the AuNRs aspect ratio. The color and UV-visible absorption spectrum of the mixed solution showed a linear relationship with the content of AP goat anti-mouse IgG enzyme-labeled secondary Ab, indicating the feasibility of developing a visual multicolor ELISA.

### 3.6. Standard Curve of AuNPs Growth-Based Multicolor ELISA for ZEN Detection

**Figure 5:**
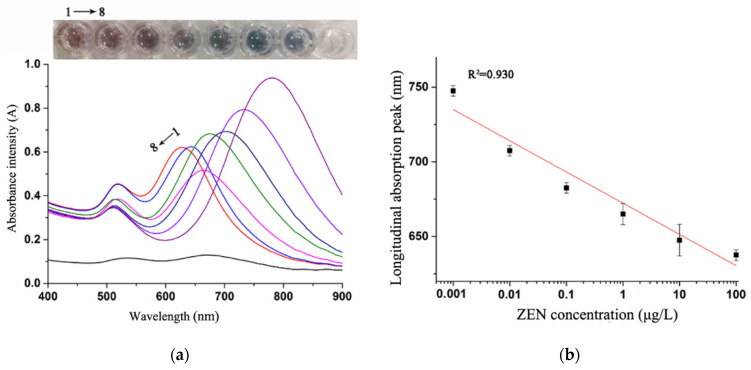
(**a**) UV absorption spectra and visual results of AuNR growth-based multicolor ELISA; (**b**) Standard curve of different ZEN concentration and AuNR longitudinal absorption peak.

The results of multicolor ELISA based on AuNR growth for ZEN at different concentrations (0–200 μg/L) are illustrated in Figure 5. The mixed solution without the addition of ZEN standard had a distinct brownish red color. As the content of ZEN increased, the color of the mixture became lighter, from dark gray to green to colorless. When the ZEN concentration was 0.1 μg/L, the mixed solution showed a color change that can be recognized by the naked eye (from brownish red to dark gray), which meant the limit of detection (LOD) of the developed AuNRs growth-based multicolor ELISA for ZEN was lower than 0.1 μg/L. The UV absorption spectra showed that the longitudinal absorption peak of AuNRs shifted to blue gradually with the increase in ZEN concentration. (Figure 5a). As shown in Figure 5b, ZEN concentration had a good linear correspondence with the longitudinal absorption wavelength of the corresponding AuNRs in the range of 0.001–100 μg/L, which strongly demonstrated the potential applicability of the developed visual multicolor ELISA based on AuNRs growth for naked identification of low concentrations of mycotoxins.

## 4. Conclusions

This study developed two detection strategies for the ZEN toxin based on the specific reaction of antigen–antibody. The established ic-ELISA method had high accuracy, sensitivity, and specificity, and can provide an effective semiquantitative analysis strategy for ZEN contaminants in food. The visual analysis strategy based on the growth of AuNRs was suitable for the rapid screening of a large number of contaminated samples ZEN and can be extended to the monitoring and control of the content of other harmful substances in foods. In Table 2, various reported ZEN detection strategies are illustrated to demonstrate the merits of the developed two methods in this study.

## Figures and Tables

**Table 1:** Cross-reacting of ZEN with other mycotoxins.

Determinand	Structure	IC_50_ (μg/L)	Cross-Reaction Rate
Zearalenone	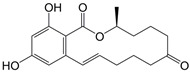	0.85	100
α-Zearalanol	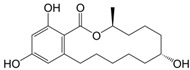	2.41	35.27
β-Zearalanol	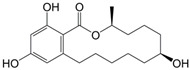	1.86	45.70
α-Zearalenol	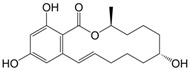	2.86	29.72
β-Zearalenol	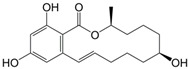	4.74	17.93
Zearalanone	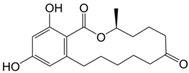	53.79	1.58
AFB_1_	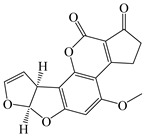	>1000	<0.01
OTA	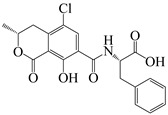	>1000	<0.01
FB_2_	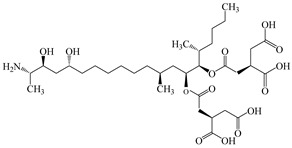	>1000	<0.01
T-2 toxin	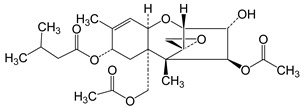	>1000	<0.01

**Table 2:** Comparison of the published traditional instrumental and immunoassays for ZEN detection.

Method Applied	Materials	Limit of Detection	References
GC-MS	-	5 ng/g	[15]
LC-MS/MS	-	0.02 ng/mL	[18]
Lateral flow immunochromatographic assays	Colloidal gold, quantum dots, polystyrene microspheres	10, 1, 1 μg/L	[22]
Fluorescence-linked immunosorbent assay	CdTe/CdS/ZnS quantum dots	0.012 ng/mL	[27]
Multiplexed immunochromatographic assay	Dual CdSe/ZnS quantum dot nanobeads	10 ng/mL	[31]
ic-ELISA	-	0.22 ± 0.08 μg/L (*IC*_15_)	This work
Visualized multicolor ELISA	AuNRs	0.1 μg/L	This work

## Data Availability

The datasets generated for this study are available on request to the corresponding author.

## Supplementary Materials

The following are available online at https://www.mdpi.com/article/10.3390/foods10112654/s1, Figure S1: Mass spectrum of ZEN-CMO, Table S1: Results of ZEN-OVA binding to Abs, Table S2: Optimization results of the coating-antigen and Ab, Table S3: Optimization results of the blocking buffer, and Table S4: Optimization results of pH value of PBS buffer.
