# Supplementary material for: Development of Indirect Competitive ELISA and Visualized Multicolor ELISA Based on Gold Nanorods Growth for the Determination of Zearalenone
Source: Foods. 2021 Nov 2;10(11):2654. doi: 10.3390/foods10112654 (PMC8619891; doi:10.3390/foods10112654)
Supplement: Supplementary file 1 [file foods-10-02654-s001.zip › foods-1431056-supplementary.pdf]

Supporting Materials

Development of Indirect Competitive ELISA and Visualized Multicolor ELISA based on Gold Nanorods Growth for the Determination of Zearalenone

Kaixin Liu [1,2], Tianyu Ma [1,2], Xiao Yang [1,2], Jingying Yang [1,2], Mingfei Pan [1,2,*] Shuo Wang [1,2]

[1] State Key Laboratory of Food Nutrition and Safety, Tianjin University of Science & Technology, 300457 Tianjin, China; Liukx2019@163.com (K.L.); maty1128@126.com (T.M.); yangx2021@126.com (X.Y.); yangjy0823@126.com (J.Y.); pmf2006@sina.com.cn (M.P.); s.wang@tust.edu.cn (S.W.).

[2] Key Laboratory of Food Nutrition and Safety, Ministry of Education of China, Tianjin University of Science and Technology, Tianjin 300457, China

* Corresponding author: Mingfei Pan

Tel: (86 22) 60912493

Fax: (86 22) 60912493

E-mail: pmf2006@sina.com.cn

Figure S1. Mass spectrum of ZEN-CMO.

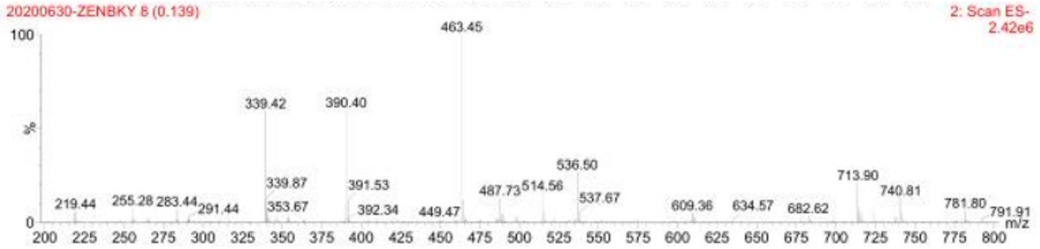

Table S1. Results of ZEN-OVA binding to Abs

	Coating amount (μg/well)	Ab dilution times	ZEN Standard Concentration (μg/L)	OD_{450}	Inhibition (%)
ZEN-OVA	0.05	16000	100	1.253	98.92

Table S2. Optimization results of the coating-antigen and Ab

Coating amount of ZEN-OVA (μg/well)	Ab dilution times	OD_{450}	IC_{50} (μg/L)
0.1	64000	0.858	2.68
0.05	32000	1.078	1.06
0.025	32000	0.793	0.85

Table S3. Optimization results of the blocking buffer

Blocking solution	OD_{450}	IC_{50} (μg/L)
0.5% skimmed milk powder	0.897	0.85
1% skimmed milk powder	1.248	2.41

Table S4. Optimization results of pH value of PBS buffer

pH of PBS diluent	OD_{450}	IC_{50} (μg/L)
5.7	0.872	2.95
7.4	1.119	0.85
8.5	0.835	1.86
